# *Cryptosporidium* spp. infection in Iranian children and immunosuppressive patients: A systematic review and meta-analysis

**DOI:** 10.22088/cjim.9.2.106

**Published:** 2018

**Authors:** Narges Kalantari, Salman Ghaffari, Masomeh Bayani

**Affiliations:** 1 Cellular and Molecular Biology Research Center, Health Research Institute, Babol University of Medical Sciences, Babol, Iran.; 2 Department of Parasitology and Mycology, Faculty of Medicine, Babol University of Medical Sciences, Babol, Iran.; 3 Infectious Diseases and Tropical Medicine Research Center, Babol University of Medical Sciences, Babol, Iran.

**Keywords:** Children, *Cryptosporidium* spp., Cryptosporidiosis, Immunosuppressive patients, Iran, Prevalence

## Abstract

**Background:**

Cryptosporidiosis is an important cause of diarrhea in children and immunosuppressive patients. The current study was intended to evaluate the prevalence rate of* Cryptosporidium* infection and clarify the epidemiological characteristics of the infection in both children and immunosuppressive patients in Iran.

**Methods:**

Five English electronic databases including PubMed, Google Scholar, Science Direct, Scopus and Cochrane, and two Persian language databases Magiran and Scientific Information Database were searched. Additionally, reports from the Iranian congresses of parasitology and graduate student thesis dissertations were assessed manually.

**Results:**

Out of 1856 studies from the literature search, our search resulted in a total of 27 articles published from 1991 to 2016. These include 14 reports on cryptosporidiosis in children and 13 papers regarding immunosuppressive patients. 8520 children and 2015 immunosuppressed cases were evaluated. Oocysts of *Cryptosporidium* were found in 3.8% and 8% children cases and immunosuppressed patients, respectively. There was a relatively high variation in the prevalence estimates among different studies, and the Q statistics was high among articles regarding children (p<0.0001) and also between records regarding immunosuppressed patients (p<0.0001). Findings showed that the prevalence rates of *Cryptosporidium* infection are significantly higher in children under 5 years (P=0.00).

**Conclusions:**

In summary, the present study provides a comprehensive view of the epidemiology of *Cryptosporidium* in children and immunosuppressive patients in Iran. Furthermore, a multidisciplinary and multicenter study to evaluate the real prevalence of *Cryptosporidium* infection and to determine its risk factors using an adequate sample size and standardized methods is highly recommended.

Cryptosporidiosis refers to the disease caused by several species of obligate, intracellular protozoan parasites known as *Cryptosporidium*. All species are able to infect the microvilli border of the gastro-intestines and respiratory epithelium of a variety of vertebrate hosts, including humans (1). A wide spectrum of clinical symptoms are observed in infected individuals because the pathogenicity of *Cryptosporidium* varies in relation with the species of parasites involved along with the age, and immune status of the host (2). The main symptoms and signs are watery diarrhea, abdominal cramps, anorexia, weight loss, nausea, vomiting, fatigue and low-grade fever (2, 3). Cryptosporidiosis affects several groups of humans including children under five years and immunocompromised individuals, particularly HIV-infected patients (4). 

Generally, this infection occurs following oocyst ingestion through the fecal–oral route. However, transmission of *Cryptosporidium* is complex and may occur from host to host (animal to human, person-to-person); through the ingestion of contaminated water or food and possibly airborne. *Cryptosporidium* produces resistant oocysts, like many other parasites which are passed in the feces into the environment. These parasites have mechanical vectors such as insects or even birds which play a role in the transmission cycle (5, 6). Until now, thirty-one Cryptosporidium species have been recognized as valid, and of these, the most common species reported in humans throughout the world are C. parvum and C. hominis (1)*.* This infection is highly prevalent and several factors contribute to the distribution of the parasite. These include shedding large numbers of oocysts from infected cases (7), resistance to the concentration and exposure times of disinfectants commonly used in the drinking water industry (5, 8), highly infectious oocysts (9) and the lack of treatment options (1).

However, the prevalence and distribution of *Cryptosporidium* spp. infection in humans differs in geographic regions of the world and also within a country. In industrialized countries, the prevalence rate of cryptosporidiosis ranges from 0.1- 9.1% (10) and the most prevalent species is *C. hominis* (11, 12). In some developing countries, the prevalence rate of *Cryptosporidium* infection ranges from 2.98-25.9% and the most frequent species is *C. hominis *(13). In Iran, the range of cryptosporidiosis is 0.3 in children (14) to 26.7% in immunosuppressive patients (15). 

**Nevertheless**, there are some publications on cryptosporidiosis in Iran but no systematic review and *meta-*analysis is available to describe the status of *Cryptosporidium* infection in this country. The current systematic review and *meta*-analysis was intended to evaluate the weighted prevalence of* Cryptosporidium* infection and clarify the epidemiological characteristics of the infection in both children and immunosuppressive patients in Iran. 

## Methods

**Search strategy:** To evaluate the epidemiological status of *Cryptosporidium* infection in humans in Iran, we designed a systematic review based on English and Persian literature released online articles published from 1991 to 2016. English electronic databases including PubMed, Google Scholar, Science Direct, Scopus and Cochrane, and two Persian language databases, Magiran and Scientific Information Database were searched. Additionally, reports from the Iranian parasitology congresses and graduate student dissertations were assessed manually. The current review was performed using medical subject headings (MeSH) terms including: “*Cryptosporidium*”, “Cryptosporidiosis”, “Prevalence”, “Epidemiology”, “Iran”, “Children” and “Immunosuppressive patients” alone or combined together.

**Study selection:** All cross-sectional studies which estimated the prevalence of *Cryptosporidium* infection and cryptosporidiosis in children and immunosuppressive patients (HIV, severe kidney failure, cancers and multiple sclerosis patients) in the Iranian population were assessed in the current review. The criteria for the diagnosis of *Cryptosporidium* infection were based on staining, serological and molecular methods. All identified studies were imported to EndNote software to remove duplicates and were also independently assessed for eligibility and inclusion by both authors. 

**Figure 1:**
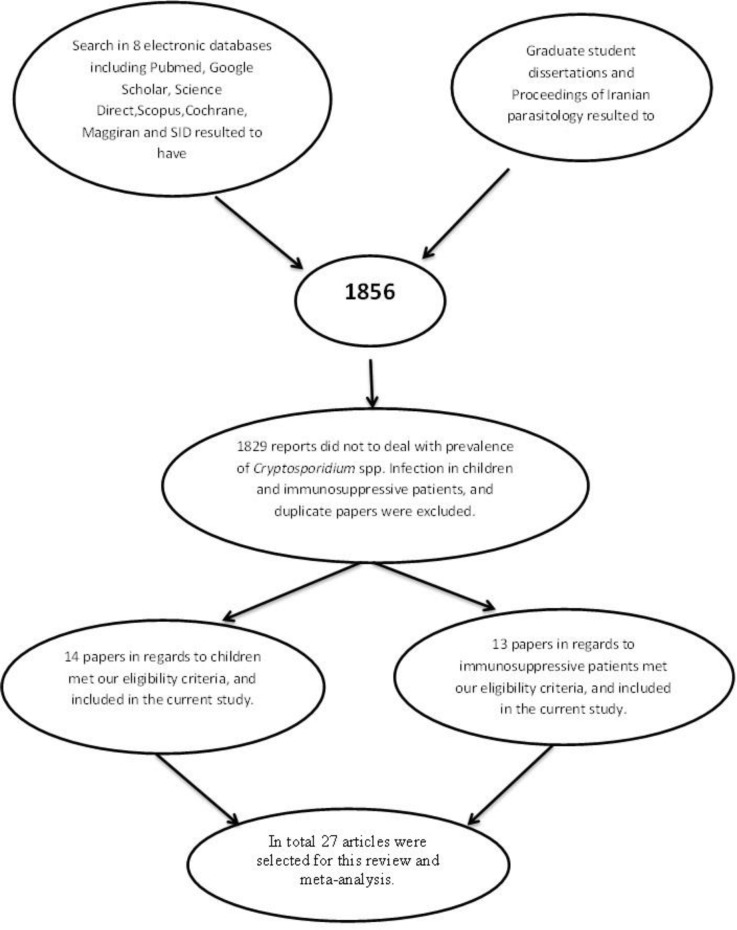
Flow diagram describing the study design process.

Figure 1 shows how the studies were categorized and the reasons for exclusion. A total of 27 studies met the present study criteria, out of 1856 that were published as journal articles and presented in national conference proceedings and graduate student dissertations. 

**Data extraction:** All articles which met the criteria for inclusion were carefully investigated and information on population, sample size, age distribution, gender, *Cryptosporidium* diagnostic methods, and year of publication, study location, and number of subjects with positive test results were withdrawn using a data extraction form. Furthermore, data on human risk factors such as pet ownership, close contact with animals, occupational group, fruit or vegetable consumption, fruit and vegetable washing methods, place of residence, and educational level was collected.

**Statistical analysis:** The prevalence in total and specific groups was calculated by age group, gender, residency and geographical region. In order to assess heterogeneity among the studies, a forest plot was used. The heterogeneity and quantifying variations were performed by statistical methods, I^2^ and Cochrane Q-statistics for the purpose of meta-analysis. A random effects model was employed as it assumed that the included studies were a random sample from a population of studies. The aforementioned plot presented proportions of individual studies and overall prevalence. The trial version of StatsDirect statistical software was used to perform this meta-analysis (www.statsdirect.com).

## Results

Out of 1856 studies from the literature search, 14 articles regarding children and 13 records about immunosuppressed patients were considered appropriate for inclusion in this systematic review and meta-analysis. Table 1 shows the results of the literature search on demographic data associated with the prevalence of *Cryptosporidium* infection in Iranian children. In total, 8520 child cases and 2015 immunosuppressed individuals were included in the current study. 

**Figure 2:**
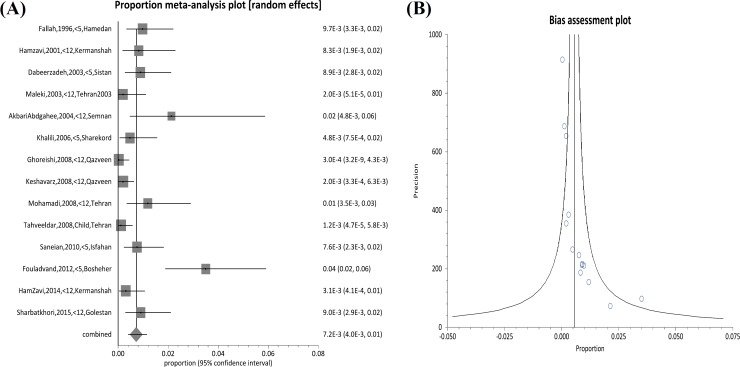
Forest plot diagram (A) and bias assessment graph (B) of 14 studies showing the prevalence rates of *Cryptosporidium *infection in Iranian children (first author, year and province of study).

**Figure 3:**
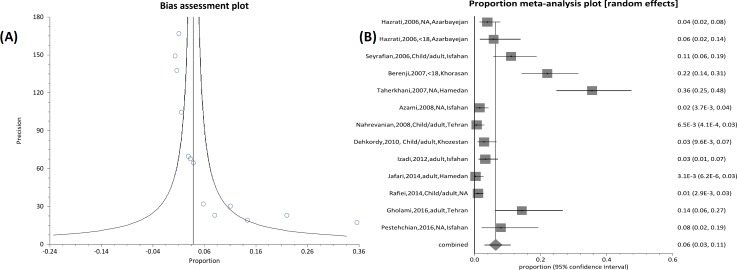
Forest plot diagram (A) and bias assessment graph (B) of 13 studies showing the prevalence rates of *Cryptosporidium *infection in Iranian immunosuppressive patients (first author, year and province of study).

Oocysts of *Cryptosporidium* were found in 294 and 125 of child cases and immunosuppressed patients, respectively. There was a relatively high variation in the prevalence estimates among the different studies, and the Q statistic was high among articles regarding children (Q=51.87, df=14, p<0.0001; I2= 74.9% (95% CI=54.1% to 83.9%) and also between records regarding immunosuppressed patients (Q=151.9, df=12, p<0.0001; I2=92.1% (95% CI=88.8% to 94.1%)) (figures 2, 3).

Modified Ziehl-Neelsen technique and enzyme-linked immunosorbent assay (ELISA) were used in the included studies. Molecular methods were also used in some studies to confirm the results of the Ziehl-Neelsen technique or to genotype the parasite. Results of the meta-analysis showed that the prevalence rates of *Cryptosporidium* infection in children under 5-years old are higher than children over 5-years old (4.21% versus 1.22%, respectively; P=0.000). Considering stool form, positive rate in the diarrheal stool and both diarrheal and non-diarrheal cases was 4.03% and 1.1% (P=0.000). The results of the literature search on demographic data associated to the prevalence of *Cryptosporidium* infection in Iranian children are shown in table 1. The results of this meta-analysis showed that the average of *Cryptosporidium* infection rate was higher in immunosuppressive cases (8%), in comparison with children (3.8%) (P= 0.038, 95% CI= -8.856041 to 0.460436). A possible association between *Cryptosporidium *infection and gender was evaluated in 10 out of 27 studies. The mean and standard deviation of prevalence in males and female cases were 5.52%±2.5 and 4.72±3.2, respectively (P=0.5). In the children group, the mean and standard deviation of prevalence was 4.83±2.2 in males and 3.52±1.4 in females (P=0.29). In the immunosuppressive patient group, the mean and standard deviation of prevalence rates were 6.22±2.8 and 5.92±4.2 (P=0.9) in male and female cases, respectively.

**Table 1:** Demographic data in relation to prevalence of* Cryptosporidium* infection in Iranian children.

**Variables**	**Participants**	**Positive cases**	**Prevalence**	**P-value**	**References**
**Gender**					
Male Female	2001 1629	77 84	3.85 5.2	0.062	28, 44, 46, 47, 49, 51, 52
**Age**					
<5 >5	3495 982	147 12	4.21 1.22	0.000	14, 28, 44,45, 46, 47,49, 51, 52
**Form of stool**					
Diarrheic Non-diarrheic	2456 798	99 9	4.03 1.1	0.000	14, 28, 44,45, 46, 47,49, 51, 52
**Residency**					
Urban Rural	878 493	12 6	1.4 1.2	0.515	14, 49
**Animal contact**					
Yes No	86 685	10 11	11.6 1.6	0.000	46, 47
**Seasons**					
Spring Summer Autumn Winter	283 178 287 156	24 11 35 8	8.5 6.2 12.2 5.1	0.038	28, 44

## Discussion

Human cryptosporidiosis is an important zoonotic infection that causes diarrhea in immunocompromised individuals and children. It also causes extra-intestinal infection in severe immunodeficiency (2). This systematic review and meta-analysis study gives a general estimate for the prevalence of *Cryptosporidium* in children and immunosuppressive patients in Iran. The overall prevalence obtained in the aforementioned population was relatively high (6%). The prevalence rates of the infection with regard to children and immunosuppressive cases were 3.8% and 8%, respectively. The total prevalence (6%) was approximately similar to some studies conducted in Malaysia (5.2%) (16), South Africa (5.59%) (17). Higher rates of infections have been reported in other studies such as in Afghanistan (14.1%) (18), Palestine (11.5%) (19), Jordan (8.3%) (20), India (12%) (21), Saudi Arabia (11%) (22) and Pakistan (10.9%) (23); and the lower rate of infection has been reported in Kenya (4%) (24) and North-West Ethiopia (4.6%) (25). Moreover, the prevalence rates of *Cryptosporidium* infection have been reported as less than 1 percent to more than 30 percent worldwide (26). In 2012, Fletcher et al. reviewed the relative prevalence of *Cryptosporidium *spp. in several developed countries and revealed that the relative prevalence rate of this infection ranges from 0.1% to 9.1% of cases. They also reported that *Cryptosporidium* infection is responsible for about 20% of diarrheal episodes in children in developing countries and up to 9% of episodes in developed regions (10). In fact, global* Cryptosporidium* infection distribution has been associated with various risk factors but its risk factors in developing nations are very different to those in industrialized countries (3). These risk factors include situation of sanitation, health status, exposure to pets and animals, nutritional behavior (consumption of raw vegetables and contaminated drinking water), unsafe sexual activity, geographical climate, and location of residence (3, 27). 

Our study results reveal that there is a high prevalence rate of* Cryptosporidium* infection in children (13.1%) in Bushehr (28), and immunosuppressive patients (26.7%) in Hamedan (15). It also demonstrated low prevalence of this infection in children (0.3%) in Qazveen (14), and immunosuppressive patients (0.56%) in Hamedan (29). A disagreement in the prevalence of *Cryptosporidium* infection in immunosuppressive patients is seen in Hamedan. A possible explanation is the type of the studied population and time. Taherkhani et al., 2007 studied HIV patients and Jafari et al., in 2014 evaluated renal transplantation cases. However, the prevalence of *Cryptosporidium* infection, like other intestinal infections, is closely related to cultural, environmental, social and economic factors (30).

Our data shows that *Cryptosporidium* prevalence in children under five years old (4.21%) was significantly higher than children above five years old (1.2%). These findings are supported by various studies which demonstrate that *Cryptosporidium *infections occur frequently in children under than 5 years where the peak of infections and diarrhea appear in children younger than 2 years old (31, 32). 

In regard to diarrhea in 4/14 studies evaluated, *Cryptosporidium *infections in both diarrheic and non-diarrheic children found that the prevalence of this infection are significantly higher in diarrheic cases. Overall, the statistical analysis of 9 studies showed that the prevalence rate of *Cryptosporidium* infection was significantly higher in diarrheic children compared with non-diarrheic children (table 1). These findings are in line with several studies, indicating that this parasite is one of most important causes of diarrhea-associated pathogens in children (33, 34). 

Our data showed that the *Cryptosporidium* prevalence rate is slightly higher in male cases than in female subjects and no significant difference was observed between the two sexes. A study reviewed cryptosporidiosis in developing countries and indicated that no significant differences were observed by sex in Kenya (13). The study of Painter et al., in the United States showed that the rates of cryptosporidiosis were higher among males than females in cases under15 years old. In contrast, cryptosporidiosis rates were higher among females aged ≥15 years (35).

This meta-analysis study was suffering from inadequate analysis for the prevalence of *Cryptosporidium *infections regarding contact with animals, level of education, occupation, residency, fruit or vegetable consumption, fruit and vegetable washing methods, and season. Therefore, the risk factors associated to cryptosporidiosis are not well known in Iran and indeed the roles of humans, livestock and wildlife in the *Cryptosporidium* transmission cycle remain largely unknown. 

Different methods for the detection of *Cryptosporidium *infections are used globally. The most common techniques are microscopic, immunological and molecular methods (36, 37). Our data showed that the microscopy method, acid-fast Ziehl-Neelsen (ZN), was the most common assay. Three and six studies used enzyme immunoassay test and molecular methods, respectively. The molecular assays revealed that *C. parvum* (84.4%) is the most common species found in human cases in Iran, followed by *C. hominis* (13.74). These findings are supported by some studies indicating that *C. parvum* and *C. hominis* are the most prevalent species in humans and that *C. parvum* is the dominant species (1, 38, 39). In contrast, several studies demonstrated that *C. hominis *is the most prevalent species in humans (7, 13, 40). Yet, the distribution of *cryptosporidium *species in humans is different amongst geographic regions and socioeconomic conditions. For example, both *C. hominis* and *C. parvum* are the commonly detected species in humans, in European countries and New Zealand but *C. parvum* is the dominant species found in humans in the Middle Eastern countries (7, 39). Only one study reported *C. meleagridis* in a child (one out of 16 positive cases) (Rafiei et al., 2014). *C.* *meleagridis* infection in a child in Mazandaran province, northern Iran was first reported in our previous study, focused on the molecular epidemiology of *Cryptosporidium* spp. in some developing countries and in the United Kingdom using restriction fragment length polymorphism (PCR-RFLP) and sequencing of the 18S rRNA gene (40), and PCR-RFLP and sequencing of *Cryptosporidium* oocyst wall protein (COWP) and 70-kDa heat shock protein (HSP70) genes (41).

In conclusion, to the best of our knowledge, the present study provides a comprehensive view of the epidemiology of *Cryptosporidium* in children and immunosuppressive patients in Iran.

 Immunosuppressive individuals show a two-fold higher prevalence rate than children and also *Cryptosporidium* infection is more prevalent in diarrheic patients compared with non-diarrheic cases. The current study suggests that these patients should be monitored regularly. It is also suggested that a multidisciplinary and multicenter study to evaluate the real prevalence of *Cryptosporidium* infection and to determine its risk factors using adequate sample size and standardized methods should be performed in Iran. Furthermore, the continuous monitoring of *Cryptosporidium* in surface water, livestock, wildlife, and humans using appropriate methods particularly molecular techniques would be helpful to increase our understanding of infection and transmission patterns of this parasite in Iran. 
